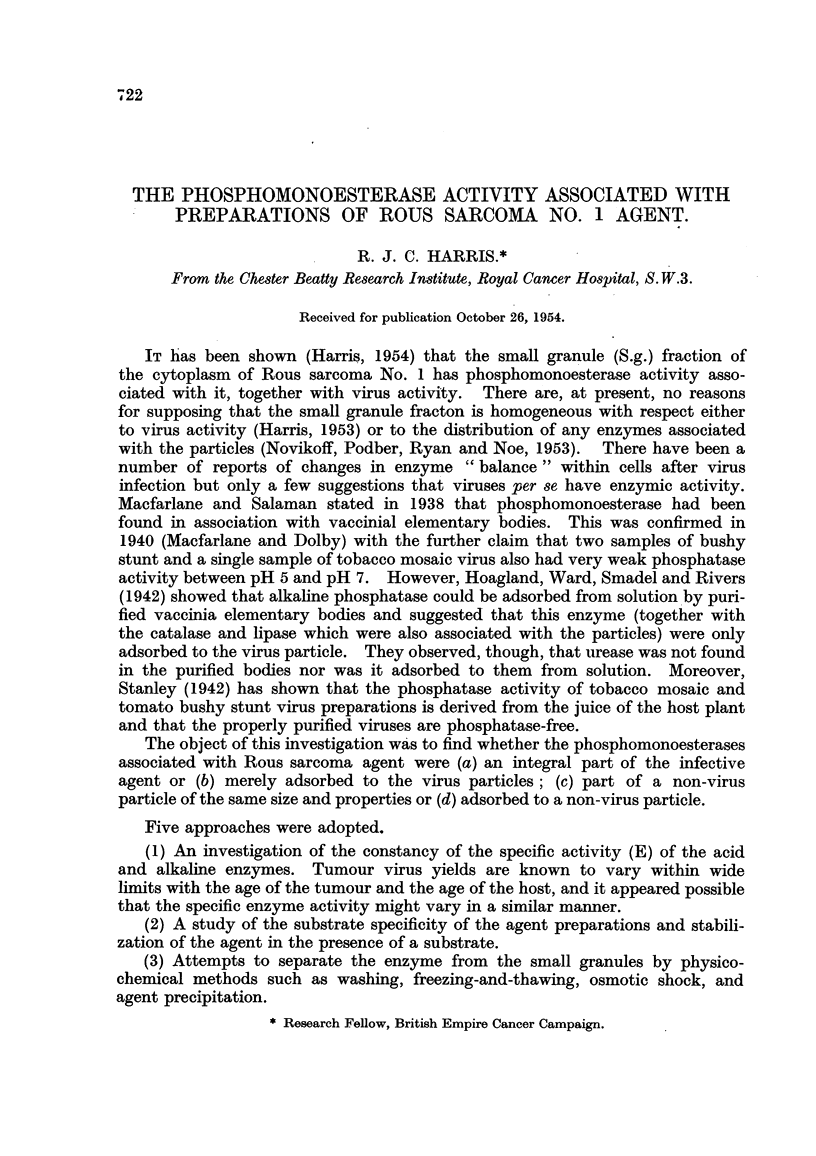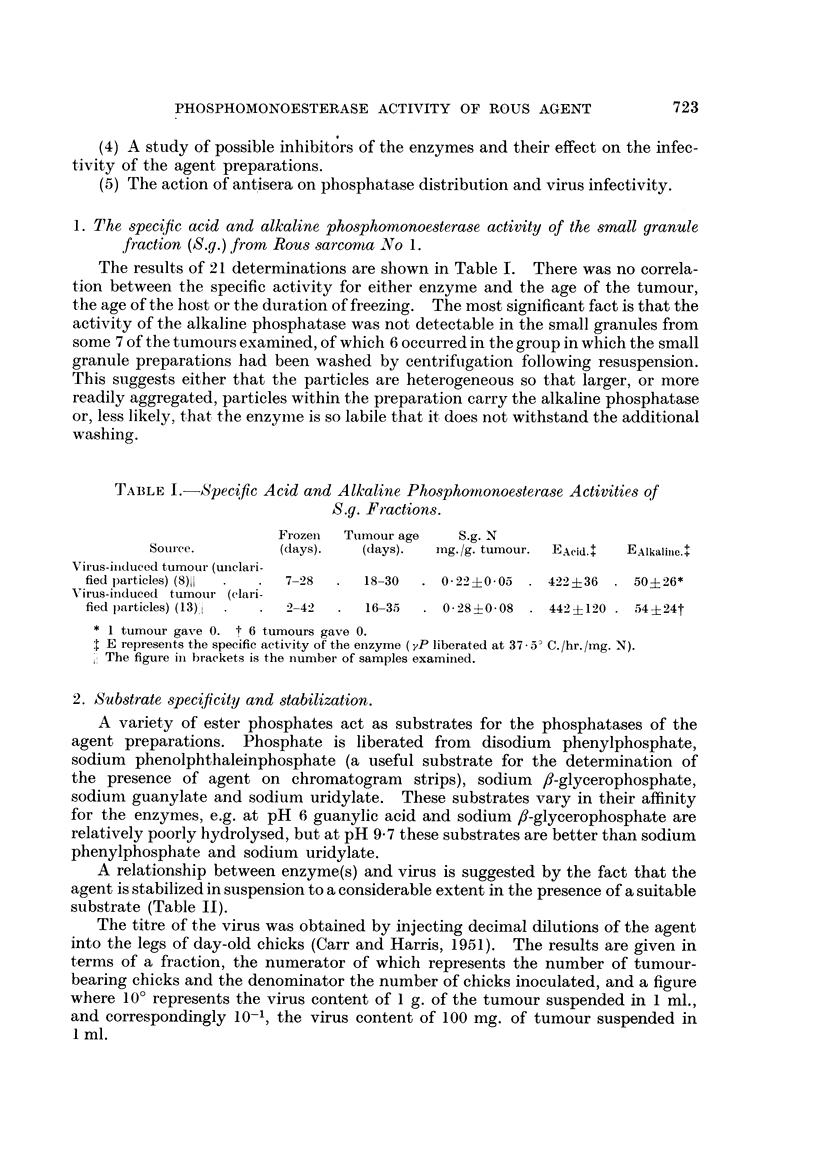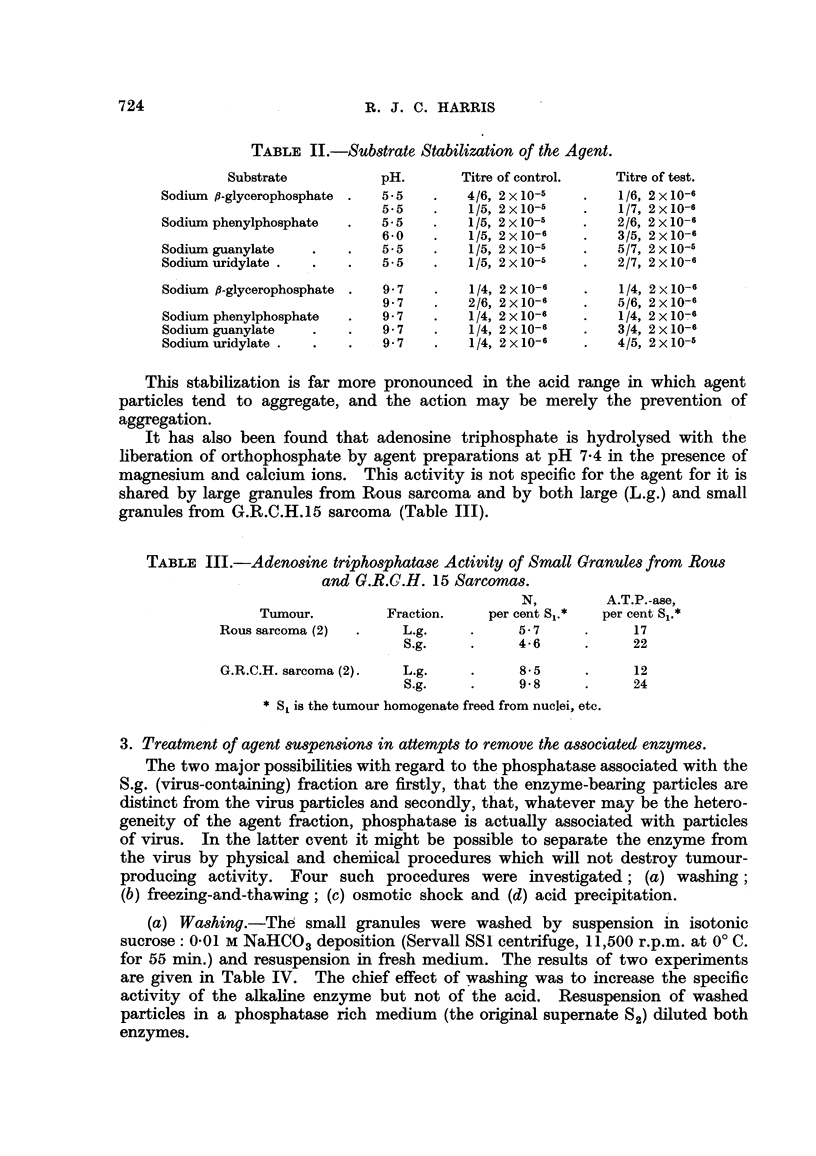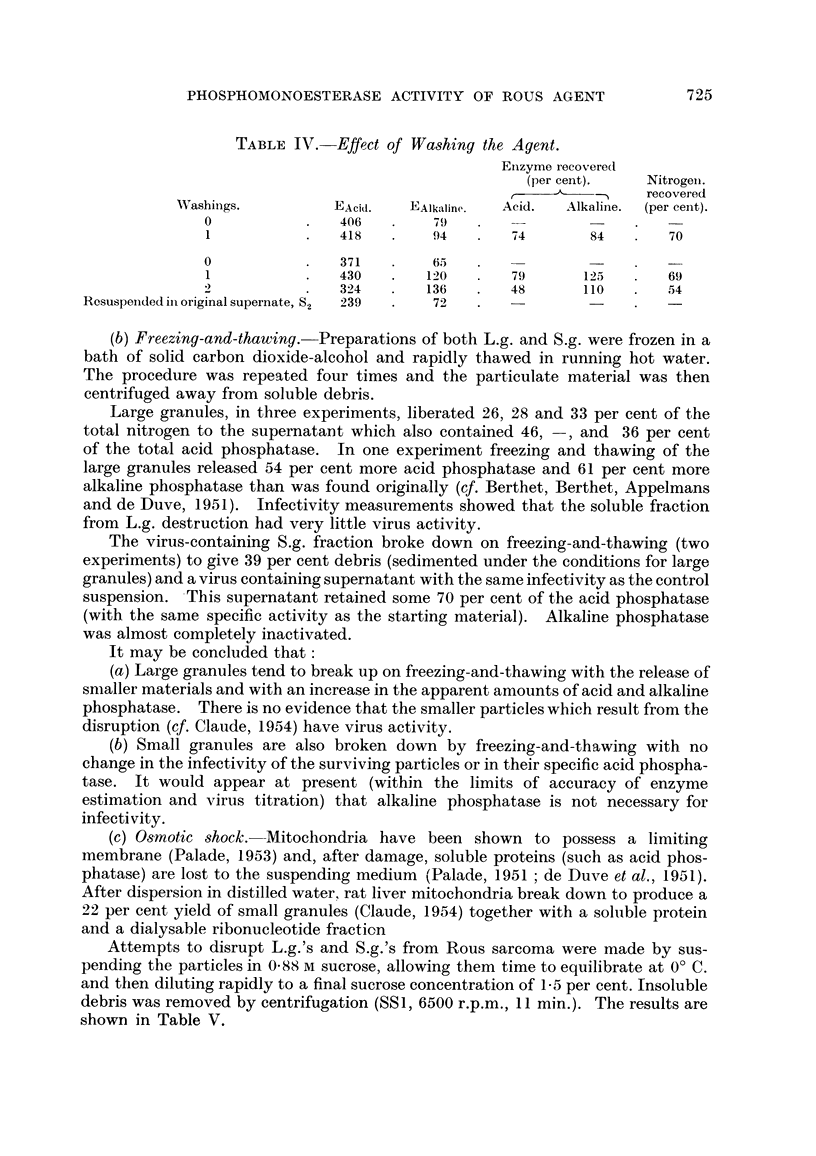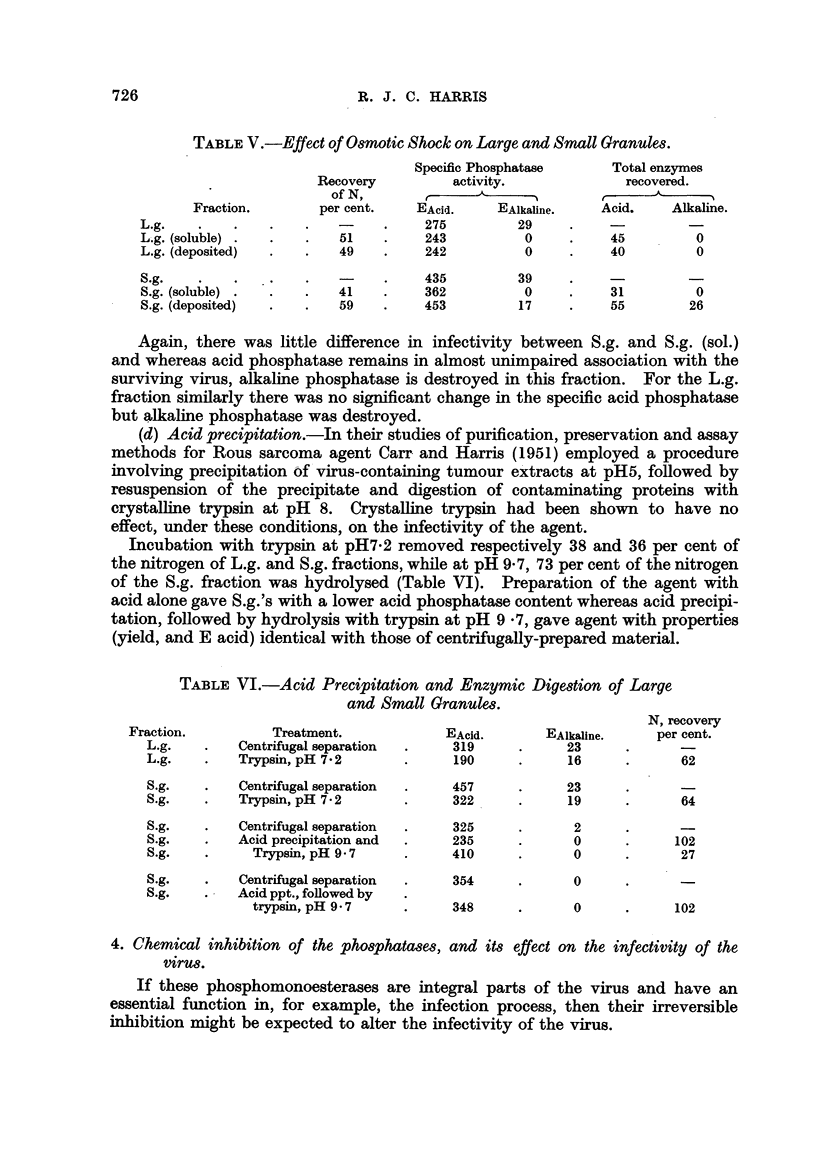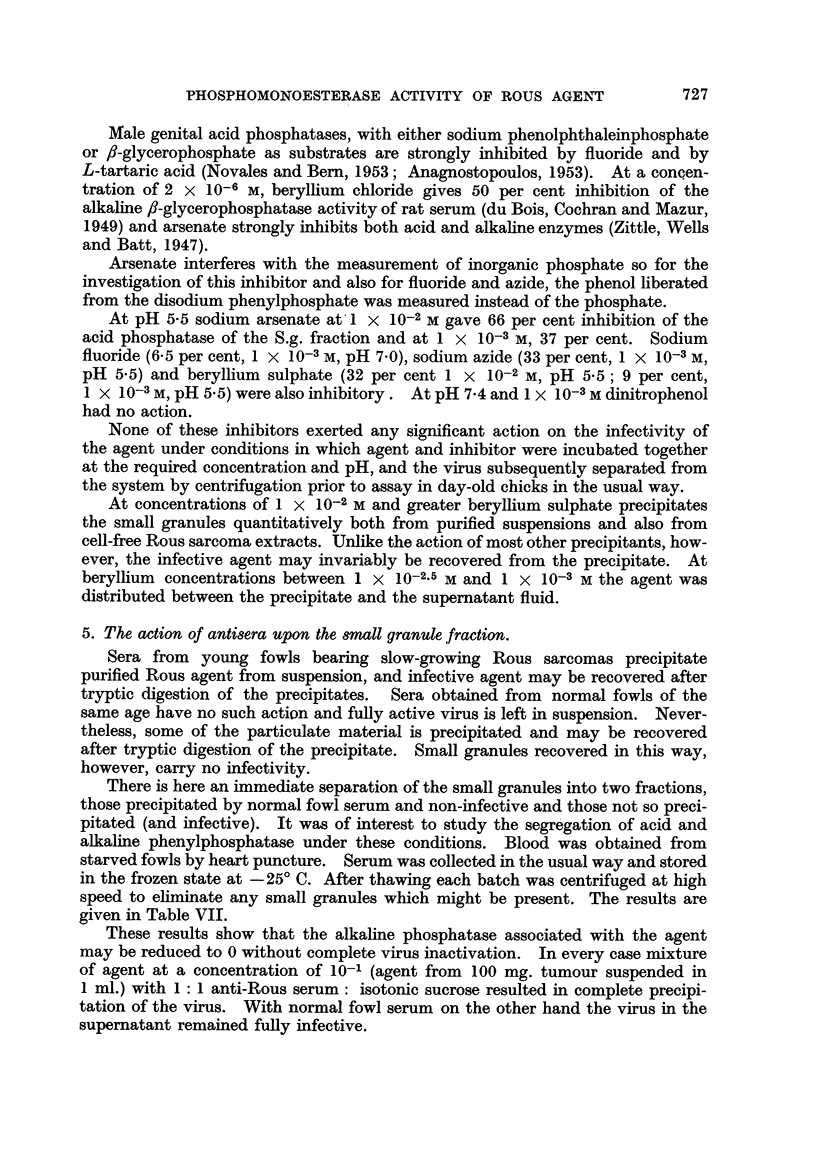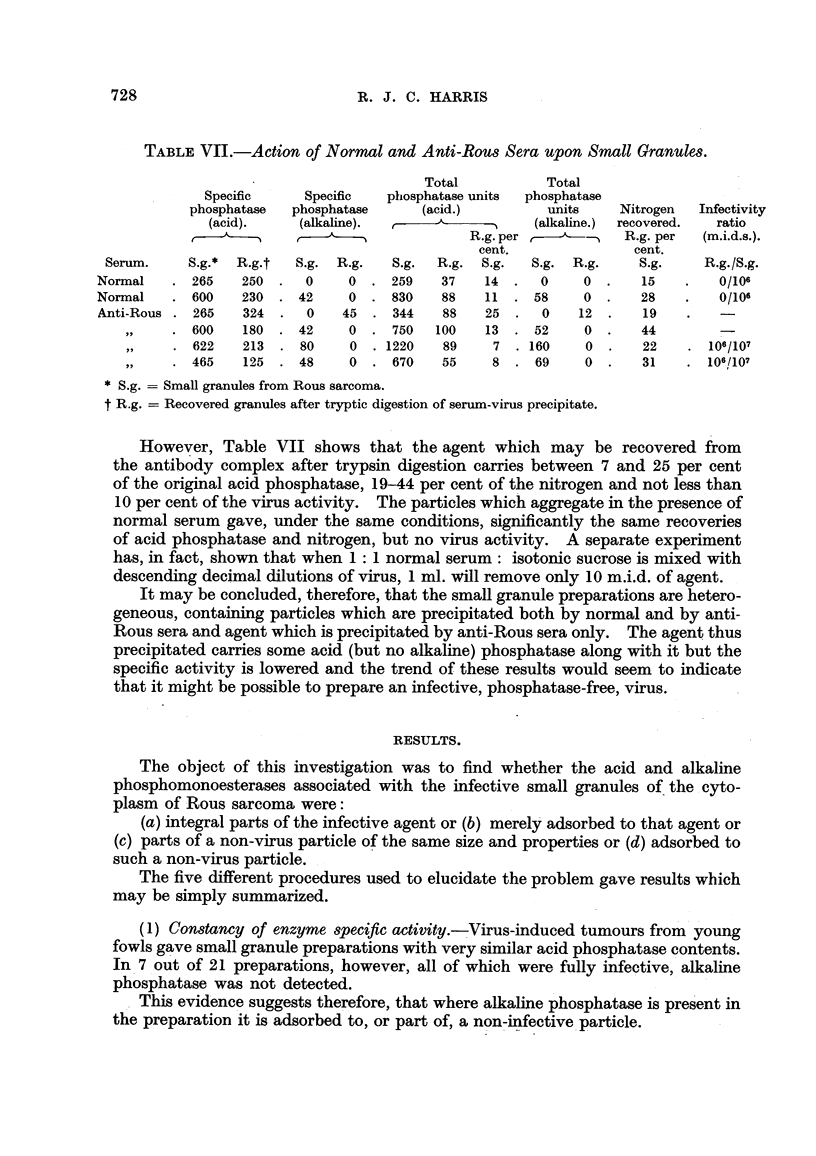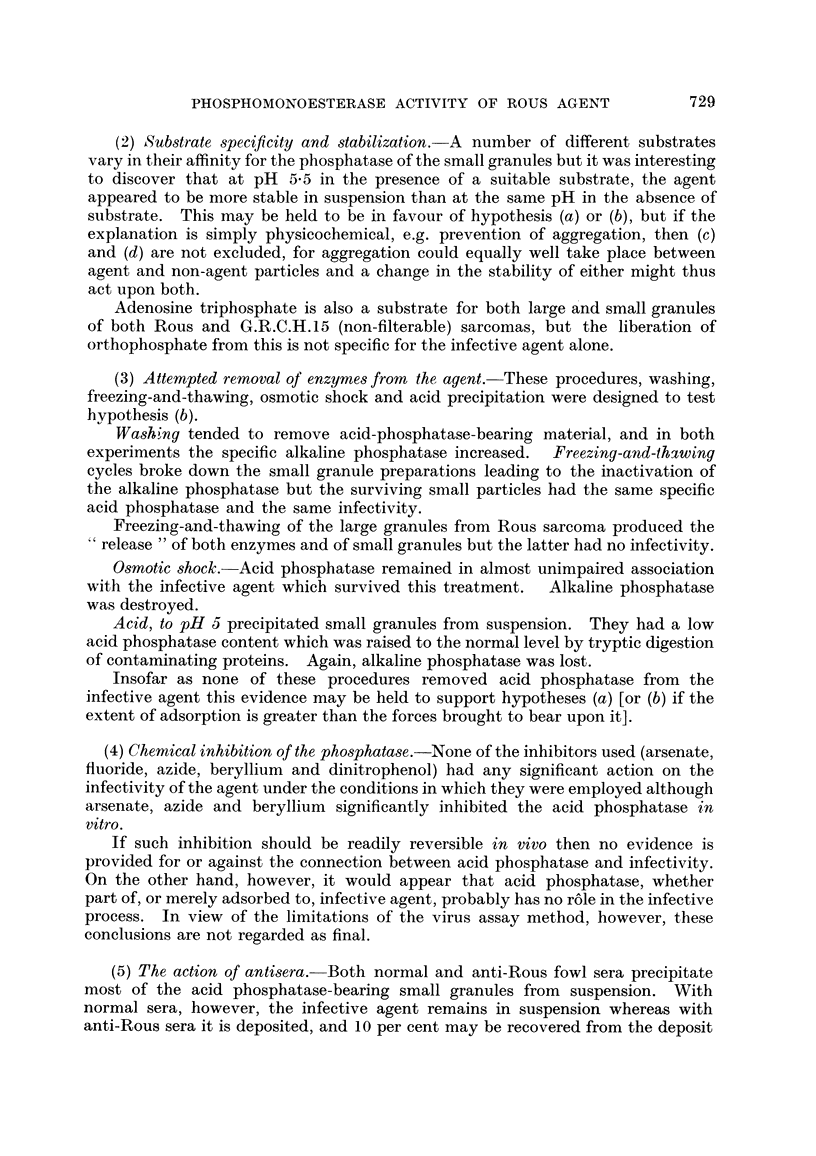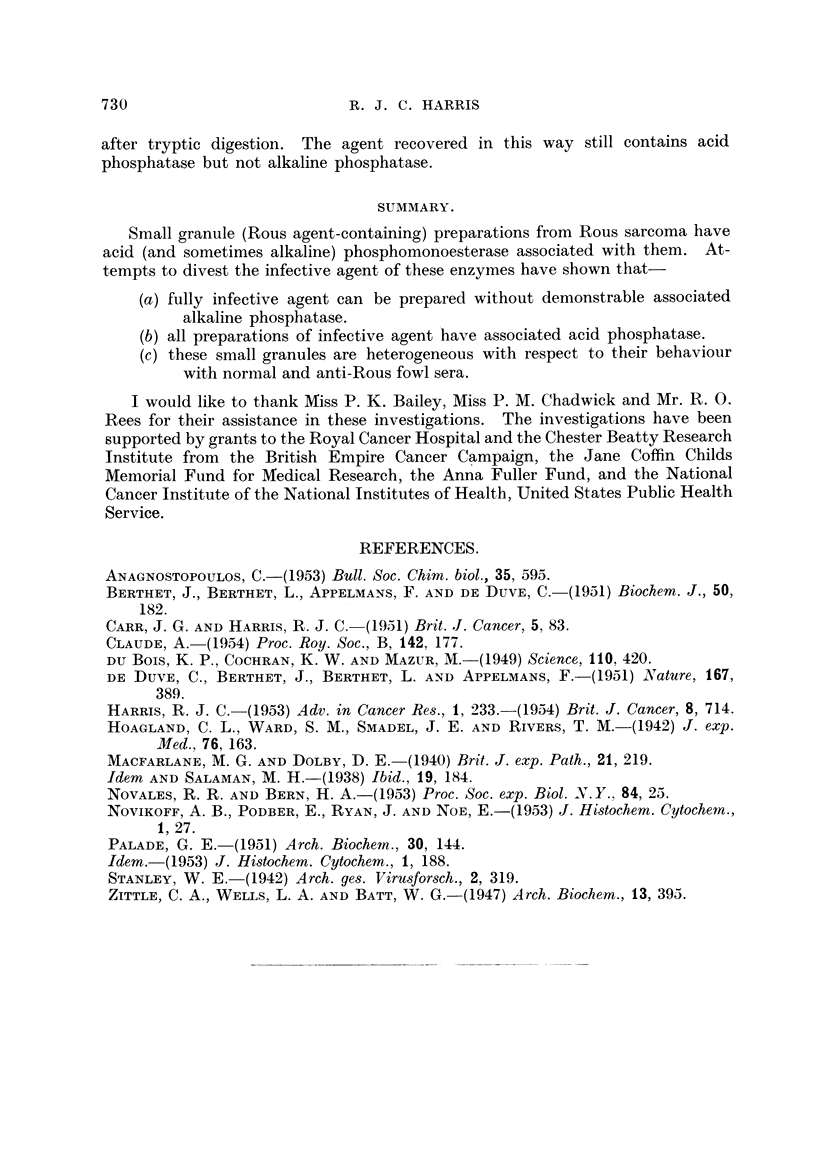# The Phosphomonoesterase Activity Associated with Preparations of Rous Sarcoma No. 1 Agent

**DOI:** 10.1038/bjc.1954.80

**Published:** 1954-12

**Authors:** R. J. C. Harris


### THE PHOSPHOMONOESTERASE ACTIVITY ASSOCIATED WITH PREPARATIONS OF ROUS SARCOMA NO. 1 AGENT.

R. J. C. HARRIS.*

*From the Chester Beatty Research Institute, Royal Cancer Hospital, S.W.3.*

Received for publication October 26, 1954.

IT has been shown (Harris, 1954) that the small granule (S.g.) fraction of the cytoplasm of Rous sarcoma No. 1 has phosphomonoesterase activity associated with it, together with virus activity. There are, at present, no reasons for supposing that the small granule fracton is homogeneous with respect either to virus activity (Harris, 1953) or to the distribution of any enzymes associated with the particles (Novikoff, Podber, Ryan and Noe, 1953). There have been a number of reports of changes in enzyme " balance " within cells after virus infection but only a few suggestions that viruses *per se* have enzymic activity. Macfarlane and Salaman stated in 1938 that phosphomonoesterase had been found in association with vaccinial elementary bodies. This was confirmed in 1940 (Macfarlane and Dolby) with the further claim that two samples of bushy stunt and a single sample of tobacco mosaic virus also had very weak phosphatase activity between pH 5 and pH 7. However, Hoagland, Ward, Smadel and Rivers (1942) showed that alkaline phosphatase could be adsorbed from solution by purified vaccinia elementary bodies and suggested that this enzyme (together with the catalase and lipase which were also associated with the particles) were only adsorbed to the virus particle. They observed, though, that urease was not found in the purified bodies nor was it adsorbed to them from solution. Moreover, Stanley (1942) has shown that the phosphatase activity of tobacco mosaic and tomato bushy stunt virus preparations is derived from the juice of the host plant and that the properly purified viruses are phosphatase-free.

The object of this investigation was to find whether the phosphomonoesterases associated with Rous sarcoma agent were (*a*) an integral part of the infective agent or (*b*) merely adsorbed to the virus particles ; (*c*) part of a non-virus particle of the same size and properties or (*d*) adsorbed to a non-virus particle.

Five approaches were adopted.

(1) An investigation of the constancy of the specific activity (E) of the acid and alkaline enzymes. Tumour virus yields are known to vary within wide limits with the age of the tumour and the age of the host, and it appeared possible that the specific enzyme activity might vary in a similar manner.

(2) A study of the substrate specificity of the agent preparations and stabilization of the agent in the presence of a substrate.

(3) Attempts to separate the enzyme from the small granules by physicochemical methods such as washing, freezing-and-thawing, osmotic shock, and agent precipitation.

* Research Fellow, British Empire Cancer Campaign.

(4) A study of possible inhibitors of the enzymes and their effect on the infectivity of the agent preparations.

(5) The action of antisera on phosphatase distribution and virus infectivity.

1. *The specific acid and alkaline phosphomonoesterase activity of the small granule fraction (S.g.) from Rous sarcoma No 1.*

The results of 21 determinations are shown in Table I. There was no correlation between the specific activity for either enzyme and the age of the tumour, the age of the host or the duration of freezing. The most significant fact is that the activity of the alkaline phosphatase was not detectable in the small granules from some 7 of the tumours examined, of which 6 occurred in the group in which the small granule preparations had been washed by centrifugation following resuspension. This suggests either that the particles are heterogeneous so that larger, or more readily aggregated, particles within the preparation carry the alkaline phosphatase or, less likely, that the enzyme is so labile that it does not withstand the additional washing.

TABLE I.—*Specific Acid and Alkaline Phosphomonoesterase Activities of S.g. Fractions.*

| Source. | Frozen (days). | Tumour age (days). | S.g. N mg./g. tumour. | $E_{Acid.}$‡ | $E_{Alkaline.}$‡ |
|---|---|---|---|---|---|
| Virus-induced tumour (unclarified particles) (8)‖ | 7–28 | 18–30 | $0 \cdot 22 \pm 0 \cdot 05$ | $422 \pm 36$ | $50 \pm 26$* |
| Virus-induced tumour (clarified particles) (13)‖ | 2–42 | 16–35 | $0 \cdot 28 \pm 0 \cdot 08$ | $442 \pm 120$ | $54 \pm 24$† |

* 1 tumour gave 0. † 6 tumours gave 0.
‡ E represents the specific activity of the enzyme ($\gamma P$ liberated at 37·5° C./hr./mg. N).
‖ The figure in brackets is the number of samples examined.

2. *Substrate specificity and stabilization.*

A variety of ester phosphates act as substrates for the phosphatases of the agent preparations. Phosphate is liberated from disodium phenylphosphate, sodium phenolphthaleinphosphate (a useful substrate for the determination of the presence of agent on chromatogram strips), sodium $\beta$-glycerophosphate, sodium guanylate and sodium uridylate. These substrates vary in their affinity for the enzymes, e.g. at pH 6 guanylic acid and sodium $\beta$-glycerophosphate are relatively poorly hydrolysed, but at pH 9·7 these substrates are better than sodium phenylphosphate and sodium uridylate.

A relationship between enzyme(s) and virus is suggested by the fact that the agent is stabilized in suspension to a considerable extent in the presence of a suitable substrate (Table II).

The titre of the virus was obtained by injecting decimal dilutions of the agent into the legs of day-old chicks (Carr and Harris, 1951). The results are given in terms of a fraction, the numerator of which represents the number of tumour-bearing chicks and the denominator the number of chicks inoculated, and a figure where $10^{0}$ represents the virus content of 1 g. of the tumour suspended in 1 ml., and correspondingly $10^{-1}$, the virus content of 100 mg. of tumour suspended in 1 ml.

TABLE II.—*Substrate Stabilization of the Agent.*

| Substrate | pH. | Titre of control. | Titre of test. |
|---|---|---|---|
| Sodium β-glycerophosphate | 5·5 | 4/6, $2\times10^{-5}$ | 1/6, $2\times10^{-6}$ |
| | 5·5 | 1/5, $2\times10^{-5}$ | 1/7, $2\times10^{-6}$ |
| Sodium phenylphosphate | 5·5 | 1/5, $2\times10^{-5}$ | 2/6, $2\times10^{-6}$ |
| | 6·0 | 1/5, $2\times10^{-6}$ | 3/5, $2\times10^{-6}$ |
| Sodium guanylate | 5·5 | 1/5, $2\times10^{-5}$ | 5/7, $2\times10^{-5}$ |
| Sodium uridylate | 5·5 | 1/5, $2\times10^{-5}$ | 2/7, $2\times10^{-6}$ |
| Sodium β-glycerophosphate | 9·7 | 1/4, $2\times10^{-6}$ | 1/4, $2\times10^{-6}$ |
| | 9·7 | 2/6, $2\times10^{-6}$ | 5/6, $2\times10^{-6}$ |
| Sodium phenylphosphate | 9·7 | 1/4, $2\times10^{-6}$ | 1/4, $2\times10^{-6}$ |
| Sodium guanylate | 9·7 | 1/4, $2\times10^{-6}$ | 3/4, $2\times10^{-6}$ |
| Sodium uridylate | 9·7 | 1/4, $2\times10^{-6}$ | 4/5, $2\times10^{-5}$ |

This stabilization is far more pronounced in the acid range in which agent particles tend to aggregate, and the action may be merely the prevention of aggregation.

It has also been found that adenosine triphosphate is hydrolysed with the liberation of orthophosphate by agent preparations at pH 7·4 in the presence of magnesium and calcium ions. This activity is not specific for the agent for it is shared by large granules from Rous sarcoma and by both large (L.g.) and small granules from G.R.C.H.15 sarcoma (Table III).

TABLE III.—*Adenosine triphosphatase Activity of Small Granules from Rous and G.R.C.H.* 15 *Sarcomas.*

| Tumour. | Fraction. | N, per cent $S_1$.* | A.T.P.-ase, per cent $S_1$.* |
|---|---|---|---|
| Rous sarcoma (2) | L.g. | 5·7 | 17 |
| | S.g. | 4·6 | 22 |
| G.R.C.H. sarcoma (2). | L.g. | 8·5 | 12 |
| | S.g. | 9·8 | 24 |

* $S_1$ is the tumour homogenate freed from nuclei, etc.

3. *Treatment of agent suspensions in attempts to remove the associated enzymes.*

The two major possibilities with regard to the phosphatase associated with the S.g. (virus-containing) fraction are firstly, that the enzyme-bearing particles are distinct from the virus particles and secondly, that, whatever may be the heterogeneity of the agent fraction, phosphatase is actually associated with particles of virus. In the latter event it might be possible to separate the enzyme from the virus by physical and chemical procedures which will not destroy tumour-producing activity. Four such procedures were investigated ; (*a*) washing ; (*b*) freezing-and-thawing ; (*c*) osmotic shock and (*d*) acid precipitation.

(*a*) *Washing.*—The small granules were washed by suspension in isotonic sucrose : 0·01 M $NaHCO_3$ deposition (Servall SS1 centrifuge, 11,500 r.p.m. at 0° C. for 55 min.) and resuspension in fresh medium. The results of two experiments are given in Table IV. The chief effect of washing was to increase the specific activity of the alkaline enzyme but not of the acid. Resuspension of washed particles in a phosphatase rich medium (the original supernate $S_2$) diluted both enzymes.

TABLE IV.—*Effect of Washing the Agent.*

| Washings. | $E_{Acid.}$ | $E_{Alkaline.}$ | Enzyme recovered (per cent). Acid. | Enzyme recovered (per cent). Alkaline. | Nitrogen. recovered (per cent). |
|---|---|---|---|---|---|
| 0 | 406 | 79 | — | — | — |
| 1 | 418 | 94 | 74 | 84 | 70 |
| 0 | 371 | 65 | — | — | — |
| 1 | 430 | 120 | 79 | 125 | 69 |
| 2 | 324 | 136 | 48 | 110 | 54 |
| Resuspended in original supernate, $S_2$ | 239 | 72 | — | — | — |

(*b*) *Freezing-and-thawing.*—Preparations of both L.g. and S.g. were frozen in a bath of solid carbon dioxide-alcohol and rapidly thawed in running hot water. The procedure was repeated four times and the particulate material was then centrifuged away from soluble debris.

Large granules, in three experiments, liberated 26, 28 and 33 per cent of the total nitrogen to the supernatant which also contained 46, —, and 36 per cent of the total acid phosphatase. In one experiment freezing and thawing of the large granules released 54 per cent more acid phosphatase and 61 per cent more alkaline phosphatase than was found originally (*cf.* Berthet, Berthet, Appelmans and de Duve, 1951). Infectivity measurements showed that the soluble fraction from L.g. destruction had very little virus activity.

The virus-containing S.g. fraction broke down on freezing-and-thawing (two experiments) to give 39 per cent debris (sedimented under the conditions for large granules) and a virus containing supernatant with the same infectivity as the control suspension. This supernatant retained some 70 per cent of the acid phosphatase (with the same specific activity as the starting material). Alkaline phosphatase was almost completely inactivated.

It may be concluded that :

(*a*) Large granules tend to break up on freezing-and-thawing with the release of smaller materials and with an increase in the apparent amounts of acid and alkaline phosphatase. There is no evidence that the smaller particles which result from the disruption (*cf.* Claude, 1954) have virus activity.

(*b*) Small granules are also broken down by freezing-and-thawing with no change in the infectivity of the surviving particles or in their specific acid phosphatase. It would appear at present (within the limits of accuracy of enzyme estimation and virus titration) that alkaline phosphatase is not necessary for infectivity.

(*c*) *Osmotic shock.*—Mitochondria have been shown to possess a limiting membrane (Palade, 1953) and, after damage, soluble proteins (such as acid phosphatase) are lost to the suspending medium (Palade, 1951 ; de Duve *et al.*, 1951). After dispersion in distilled water, rat liver mitochondria break down to produce a 22 per cent yield of small granules (Claude, 1954) together with a soluble protein and a dialysable ribonucleotide fraction

Attempts to disrupt L.g.'s and S.g.'s from Rous sarcoma were made by suspending the particles in 0·88 M sucrose, allowing them time to equilibrate at 0° C. and then diluting rapidly to a final sucrose concentration of 1·5 per cent. Insoluble debris was removed by centrifugation (SS1, 6500 r.p.m., 11 min.). The results are shown in Table V.

TABLE V.—*Effect of Osmotic Shock on Large and Small Granules.*

| Fraction. | Recovery of N, per cent. | Specific Phosphatase activity. E Acid. | E Alkaline. | Total enzymes recovered. Acid. | Alkaline. |
|---|---|---|---|---|---|
| L.g. | — | 275 | 29 | — | — |
| L.g. (soluble) | 51 | 243 | 0 | 45 | 0 |
| L.g. (deposited) | 49 | 242 | 0 | 40 | 0 |
| S.g. | — | 435 | 39 | — | — |
| S.g. (soluble) | 41 | 362 | 0 | 31 | 0 |
| S.g. (deposited) | 59 | 453 | 17 | 55 | 26 |

Again, there was little difference in infectivity between S.g. and S.g. (sol.) and whereas acid phosphatase remains in almost unimpaired association with the surviving virus, alkaline phosphatase is destroyed in this fraction. For the L.g. fraction similarly there was no significant change in the specific acid phosphatase but alkaline phosphatase was destroyed.

(*d*) *Acid precipitation.*—In their studies of purification, preservation and assay methods for Rous sarcoma agent Carr and Harris (1951) employed a procedure involving precipitation of virus-containing tumour extracts at pH5, followed by resuspension of the precipitate and digestion of contaminating proteins with crystalline trypsin at pH 8. Crystalline trypsin had been shown to have no effect, under these conditions, on the infectivity of the agent.

Incubation with trypsin at pH7·2 removed respectively 38 and 36 per cent of the nitrogen of L.g. and S.g. fractions, while at pH 9·7, 73 per cent of the nitrogen of the S.g. fraction was hydrolysed (Table VI). Preparation of the agent with acid alone gave S.g.'s with a lower acid phosphatase content whereas acid precipitation, followed by hydrolysis with trypsin at pH 9 ·7, gave agent with properties (yield, and E acid) identical with those of centrifugally-prepared material.

TABLE VI.—*Acid Precipitation and Enzymic Digestion of Large and Small Granules.*

| Fraction. | Treatment. | E Acid. | E Alkaline. | N, recovery per cent. |
|---|---|---|---|---|
| L.g. | Centrifugal separation | 319 | 23 | — |
| L.g. | Trypsin, pH 7·2 | 190 | 16 | 62 |
| S.g. | Centrifugal separation | 457 | 23 | — |
| S.g. | Trypsin, pH 7·2 | 322 | 19 | 64 |
| S.g. | Centrifugal separation | 325 | 2 | — |
| S.g. | Acid precipitation and | 235 | 0 | 102 |
| S.g. | Trypsin, pH 9·7 | 410 | 0 | 27 |
| S.g. | Centrifugal separation | 354 | 0 | — |
| S.g. | Acid ppt., followed by trypsin, pH 9·7 | 348 | 0 | 102 |

4. *Chemical inhibition of the phosphatases, and its effect on the infectivity of the virus.*

If these phosphomonoesterases are integral parts of the virus and have an essential function in, for example, the infection process, then their irreversible inhibition might be expected to alter the infectivity of the virus.

Male genital acid phosphatases, with either sodium phenolphthaleinphosphate or $\beta$-glycerophosphate as substrates are strongly inhibited by fluoride and by $L$-tartaric acid (Novales and Bern, 1953; Anagnostopoulos, 1953). At a concentration of $2 \times 10^{-6}$ M, beryllium chloride gives 50 per cent inhibition of the alkaline $\beta$-glycerophosphatase activity of rat serum (du Bois, Cochran and Mazur, 1949) and arsenate strongly inhibits both acid and alkaline enzymes (Zittle, Wells and Batt, 1947).

Arsenate interferes with the measurement of inorganic phosphate so for the investigation of this inhibitor and also for fluoride and azide, the phenol liberated from the disodium phenylphosphate was measured instead of the phosphate.

At pH 5·5 sodium arsenate at $1 \times 10^{-2}$ M gave 66 per cent inhibition of the acid phosphatase of the S.g. fraction and at $1 \times 10^{-3}$ M, 37 per cent. Sodium fluoride (6·5 per cent, $1 \times 10^{-3}$ M, pH 7·0), sodium azide (33 per cent, $1 \times 10^{-3}$ M, pH 5·5) and beryllium sulphate (32 per cent $1 \times 10^{-2}$ M, pH 5·5; 9 per cent, $1 \times 10^{-3}$ M, pH 5·5) were also inhibitory. At pH 7·4 and $1 \times 10^{-3}$ M dinitrophenol had no action.

None of these inhibitors exerted any significant action on the infectivity of the agent under conditions in which agent and inhibitor were incubated together at the required concentration and pH, and the virus subsequently separated from the system by centrifugation prior to assay in day-old chicks in the usual way.

At concentrations of $1 \times 10^{-2}$ M and greater beryllium sulphate precipitates the small granules quantitatively both from purified suspensions and also from cell-free Rous sarcoma extracts. Unlike the action of most other precipitants, however, the infective agent may invariably be recovered from the precipitate. At beryllium concentrations between $1 \times 10^{-2.5}$ M and $1 \times 10^{-3}$ M the agent was distributed between the precipitate and the supernatant fluid.

5. *The action of antisera upon the small granule fraction.*

Sera from young fowls bearing slow-growing Rous sarcomas precipitate purified Rous agent from suspension, and infective agent may be recovered after tryptic digestion of the precipitates. Sera obtained from normal fowls of the same age have no such action and fully active virus is left in suspension. Nevertheless, some of the particulate material is precipitated and may be recovered after tryptic digestion of the precipitate. Small granules recovered in this way, however, carry no infectivity.

There is here an immediate separation of the small granules into two fractions, those precipitated by normal fowl serum and non-infective and those not so precipitated (and infective). It was of interest to study the segregation of acid and alkaline phenylphosphatase under these conditions. Blood was obtained from starved fowls by heart puncture. Serum was collected in the usual way and stored in the frozen state at $-25°$ C. After thawing each batch was centrifuged at high speed to eliminate any small granules which might be present. The results are given in Table VII.

These results show that the alkaline phosphatase associated with the agent may be reduced to 0 without complete virus inactivation. In every case mixture of agent at a concentration of $10^{-1}$ (agent from 100 mg. tumour suspended in 1 ml.) with 1 : 1 anti-Rous serum : isotonic sucrose resulted in complete precipitation of the virus. With normal fowl serum on the other hand the virus in the supernatant remained fully infective.

TABLE VII.—*Action of Normal and Anti-Rous Sera upon Small Granules.*

| Serum. | Specific phosphatase (acid). S.g.* | R.g.† | Specific phosphatase (alkaline). S.g. | R.g. | Total phosphatase units (acid.) S.g. | R.g. | R.g. per cent. S.g. | Total phosphatase units (alkaline.) S.g. | R.g. | Nitrogen recovered. R.g. per cent. S.g. | Infectivity ratio (m.i.d.s.). R.g./S.g. |
|---|---|---|---|---|---|---|---|---|---|---|---|
| Normal | 265 | 250 | 0 | 0 | 259 | 37 | 14 | 0 | 0 | 15 | 0/$10^6$ |
| Normal | 600 | 230 | 42 | 0 | 830 | 88 | 11 | 58 | 0 | 28 | 0/$10^6$ |
| Anti-Rous | 265 | 324 | 0 | 45 | 344 | 88 | 25 | 0 | 12 | 19 | — |
| „ | 600 | 180 | 42 | 0 | 750 | 100 | 13 | 52 | 0 | 44 | — |
| „ | 622 | 213 | 80 | 0 | 1220 | 89 | 7 | 160 | 0 | 22 | $10^6/10^7$ |
| „ | 465 | 125 | 48 | 0 | 670 | 55 | 8 | 69 | 0 | 31 | $10^6/10^7$ |

* S.g. = Small granules from Rous sarcoma.
† R.g. = Recovered granules after tryptic digestion of serum-virus precipitate.

However, Table VII shows that the agent which may be recovered from the antibody complex after trypsin digestion carries between 7 and 25 per cent of the original acid phosphatase, 19–44 per cent of the nitrogen and not less than 10 per cent of the virus activity. The particles which aggregate in the presence of normal serum gave, under the same conditions, significantly the same recoveries of acid phosphatase and nitrogen, but no virus activity. A separate experiment has, in fact, shown that when 1 : 1 normal serum : isotonic sucrose is mixed with descending decimal dilutions of virus, 1 ml. will remove only 10 m.i.d. of agent.

It may be concluded, therefore, that the small granule preparations are heterogeneous, containing particles which are precipitated both by normal and by anti-Rous sera and agent which is precipitated by anti-Rous sera only. The agent thus precipitated carries some acid (but no alkaline) phosphatase along with it but the specific activity is lowered and the trend of these results would seem to indicate that it might be possible to prepare an infective, phosphatase-free, virus.

### RESULTS.

The object of this investigation was to find whether the acid and alkaline phosphomonoesterases associated with the infective small granules of the cytoplasm of Rous sarcoma were:

(*a*) integral parts of the infective agent or (*b*) merely adsorbed to that agent or (*c*) parts of a non-virus particle of the same size and properties or (*d*) adsorbed to such a non-virus particle.

The five different procedures used to elucidate the problem gave results which may be simply summarized.

(1) *Constancy of enzyme specific activity.*—Virus-induced tumours from young fowls gave small granule preparations with very similar acid phosphatase contents. In 7 out of 21 preparations, however, all of which were fully infective, alkaline phosphatase was not detected.

This evidence suggests therefore, that where alkaline phosphatase is present in the preparation it is adsorbed to, or part of, a non-infective particle.

(2) *Substrate specificity and stabilization.*—A number of different substrates vary in their affinity for the phosphatase of the small granules but it was interesting to discover that at pH 5·5 in the presence of a suitable substrate, the agent appeared to be more stable in suspension than at the same pH in the absence of substrate. This may be held to be in favour of hypothesis (*a*) or (*b*), but if the explanation is simply physicochemical, e.g. prevention of aggregation, then (*c*) and (*d*) are not excluded, for aggregation could equally well take place between agent and non-agent particles and a change in the stability of either might thus act upon both.

Adenosine triphosphate is also a substrate for both large and small granules of both Rous and G.R.C.H.15 (non-filterable) sarcomas, but the liberation of orthophosphate from this is not specific for the infective agent alone.

(3) *Attempted removal of enzymes from the agent.*—These procedures, washing, freezing-and-thawing, osmotic shock and acid precipitation were designed to test hypothesis (*b*).

*Washing* tended to remove acid-phosphatase-bearing material, and in both experiments the specific alkaline phosphatase increased. *Freezing-and-thawing* cycles broke down the small granule preparations leading to the inactivation of the alkaline phosphatase but the surviving small particles had the same specific acid phosphatase and the same infectivity.

Freezing-and-thawing of the large granules from Rous sarcoma produced the "release" of both enzymes and of small granules but the latter had no infectivity.

*Osmotic shock.*—Acid phosphatase remained in almost unimpaired association with the infective agent which survived this treatment. Alkaline phosphatase was destroyed.

*Acid, to pH 5* precipitated small granules from suspension. They had a low acid phosphatase content which was raised to the normal level by tryptic digestion of contaminating proteins. Again, alkaline phosphatase was lost.

Insofar as none of these procedures removed acid phosphatase from the infective agent this evidence may be held to support hypotheses (*a*) [or (*b*) if the extent of adsorption is greater than the forces brought to bear upon it].

(4) *Chemical inhibition of the phosphatase.*—None of the inhibitors used (arsenate, fluoride, azide, beryllium and dinitrophenol) had any significant action on the infectivity of the agent under the conditions in which they were employed although arsenate, azide and beryllium significantly inhibited the acid phosphatase *in vitro*.

If such inhibition should be readily reversible *in vivo* then no evidence is provided for or against the connection between acid phosphatase and infectivity. On the other hand, however, it would appear that acid phosphatase, whether part of, or merely adsorbed to, infective agent, probably has no rôle in the infective process. In view of the limitations of the virus assay method, however, these conclusions are not regarded as final.

(5) *The action of antisera.*—Both normal and anti-Rous fowl sera precipitate most of the acid phosphatase-bearing small granules from suspension. With normal sera, however, the infective agent remains in suspension whereas with anti-Rous sera it is deposited, and 10 per cent may be recovered from the deposit after tryptic digestion. The agent recovered in this way still contains acid phosphatase but not alkaline phosphatase.

### SUMMARY.

Small granule (Rous agent-containing) preparations from Rous sarcoma have acid (and sometimes alkaline) phosphomonoesterase associated with them. Attempts to divest the infective agent of these enzymes have shown that—

(*a*) fully infective agent can be prepared without demonstrable associated alkaline phosphatase.
(*b*) all preparations of infective agent have associated acid phosphatase.
(*c*) these small granules are heterogeneous with respect to their behaviour with normal and anti-Rous fowl sera.

I would like to thank Miss P. K. Bailey, Miss P. M. Chadwick and Mr. R. O. Rees for their assistance in these investigations. The investigations have been supported by grants to the Royal Cancer Hospital and the Chester Beatty Research Institute from the British Empire Cancer Campaign, the Jane Coffin Childs Memorial Fund for Medical Research, the Anna Fuller Fund, and the National Cancer Institute of the National Institutes of Health, United States Public Health Service.

### REFERENCES.

Anagnostopoulos, C.—(1953) *Bull. Soc. Chim. biol.*, **35**, 595.
Berthet, J., Berthet, L., Appelmans, F. and de Duve, C.—(1951) *Biochem. J.*, **50**, 182.
Carr, J. G. and Harris, R. J. C.—(1951) *Brit. J. Cancer*, **5**, 83.
Claude, A.—(1954) *Proc. Roy. Soc.*, B, **142**, 177.
du Bois, K. P., Cochran, K. W. and Mazur, M.—(1949) *Science*, **110**, 420.
de Duve, C., Berthet, J., Berthet, L. and Appelmans, F.—(1951) *Nature*, **167**, 389.
Harris, R. J. C.—(1953) *Adv. in Cancer Res.*, **1**, 233.—(1954) *Brit. J. Cancer*, **8**, 714.
Hoagland, C. L., Ward, S. M., Smadel, J. E. and Rivers, T. M.—(1942) *J. exp. Med.*, **76**, 163.
Macfarlane, M. G. and Dolby, D. E.—(1940) *Brit. J. exp. Path.*, **21**, 219.
*Idem* and Salaman, M. H.—(1938) *Ibid.*, **19**, 184.
Novales, R. R. and Bern, H. A.—(1953) *Proc. Soc. exp. Biol. N.Y.*, **84**, 25.
Novikoff, A. B., Podber, E., Ryan, J. and Noe, E.—(1953) *J. Histochem. Cytochem.*, **1**, 27.
Palade, G. E.—(1951) *Arch. Biochem.*, **30**, 144.
*Idem.*—(1953) *J. Histochem. Cytochem.*, **1**, 188.
Stanley, W. E.—(1942) *Arch. ges. Virusforsch.*, **2**, 319.
Zittle, C. A., Wells, L. A. and Batt, W. G.—(1947) *Arch. Biochem.*, **13**, 395.